# Effect of Isopropyl Alcohol Concentration and Etching Time on Wet Chemical Anisotropic Etching of Low-Resistivity Crystalline Silicon Wafer

**DOI:** 10.1155/2017/7542870

**Published:** 2017-07-31

**Authors:** Eyad Abdur-Rahman, Ibrahim Alghoraibi, Hassan Alkurdi

**Affiliations:** ^1^Physics Department, Damascus University, Baramkeh, Damascus, Syria; ^2^Department of Basic and Supporting Sciences, Faculty of Pharmacy, Arab International University, Damascus, Syria

## Abstract

A micropyramid structure was formed on the surface of a monocrystalline silicon wafer (100) using a wet chemical anisotropic etching technique. The main objective was to evaluate the performance of the etchant based on the silicon surface reflectance. Different isopropyl alcohol (IPA) volume concentrations (2, 4, 6, 8, and 10%) and different etching times (10, 20, 30, 40, and 50 min) were selected to study the total reflectance of silicon wafers. The other parameters such as NaOH concentration (12% wt.), the temperature of the solution (81.5°C), and range of stirrer speeds (400 rpm) were kept constant for all processes. The surface morphology of the wafer was analyzed by optical microscopy and atomic force microscopy (AFM). The AFM images confirmed a well-uniform pyramidal structure with various average pyramid sizes ranging from 1 to 1.6 *μ*m. A UV-Vis spectrophotometer with integrating sphere was used to obtain the total reflectivity. The textured silicon wafers show high absorbance in the visible region. The optimum texture-etching parameters were found to be 4–6% vol. IPA and 40 min at which the average total reflectance of the silicon wafer was reduced to 11.22%.

## 1. Introduction

**Figure 1:**
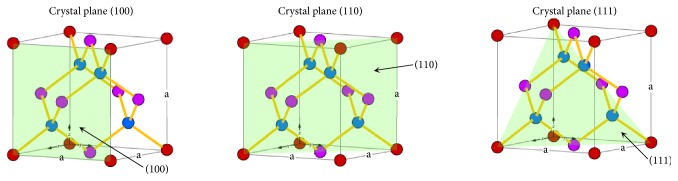
Main silicon crystal planes.

Silicon solar cells dominate the current photovoltaic market [1] due to their advantages, including low cost, easy fabrication, and environmental friendliness [2]. Planar Si surfaces have a high natural reflectivity with a strong spectral dependence [3]. In order to reduce this high reflectivity and to trap the light in the solar cell, different surface texturing techniques have been developed over the last years [4–8] like plasma etching [9, 10], mechanical engraving [10], chemical anisotropic etching [11], laser texturing [12, 13], and reactive ion etching [14, 15]. However, the wet chemical anisotropic etching in alkaline solutions is the most common process for industrial solar cell texturing [15] because it is actually a good compromise between cost and efficiency [10]. These solutions rely on the difference in etch rate between 〈100〉 and 〈111〉 oriented planes (Figure 1) and result in random, upright micrometer-scale pyramids on a 〈100〉 oriented surface. Each pyramid forces the reflected ray to be incident on an adjacent pyramid and thus to undergo another reflection into the wafer. Hence, light collection increases due to multiple internal reflections. Alkaline solutions used in anisotropic etching can be either an organic or an inorganic compound. Among all alkaline solutions, the two inorganic KOH and NaOH solutions and the organic TMAH (tetramethylammonium hydroxide) solution are the most frequently used [6]. Silicon reacts with NaOH in deionized water (DI-W) as in the following total reaction equation [7]:

(1) $$\begin{array}{c} Si_{\left(s\right)}+2NaOH_{\left(aq\right)}+H_{2}O_{\left(l\right)}⟶Na_{2}SiO_{3\left(aq\right)}+2H_{2\left(g\right)}↑ \end{array}$$

The alkaline solution etches 〈111〉 planes with a very low etching rate compared with other planes, especially 〈100〉 planes (the etching rate ratio for 〈100〉 to 〈111〉 planes is 10~35). This strong dependence of the etching rate on crystal orientation leads to the formation of 4-sided pyramidal structures that have a 〈100〉 base plane and 〈111〉 faces [16].

**Figure 2:**
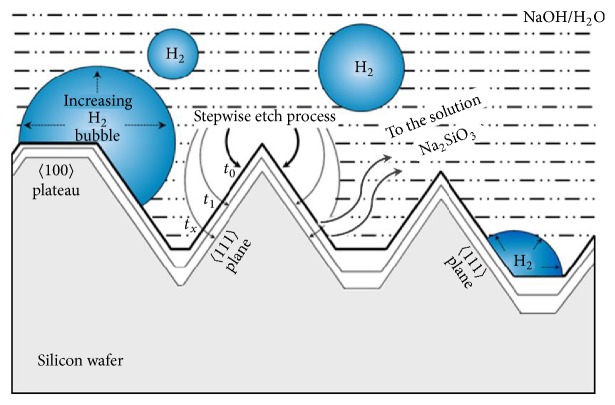
Cross-sectional view of random pyramid texturing.

One problem in the texturing process is the generation of H_2_ bubbles that attach to the wafer's surface causing the formation of big pyramids and low uniformed surface texture (Figure 2). Many studies reported that adding isopropyl alcohol (IPA) increases the wettability of the silicon surface [6] and then removes the adhering hydrogen bubbles sticking on the surface, leading to an increase in the uniformity of the random pyramids. The other effect of adding IPA is that it strongly decreases the etching rate of the silicon wafer [8, 17]. Additionally, few studies focused on the amount of IPA ensuring the improvement of the surface morphology in the etching process.

However, the topography of the Si surface also depends on a number of parameters including the concentration of the etching solution [18], the solution temperature [18], the texturing time, and the presence of a surfactant or catalyst [6]. Mechanical agitation is reported to have a significant effect on the quality of the etching process and on the etching rate [19]. This is because stirring the solution enhances the uniformity of the random pyramid texture as it drifts the reaction products away from the surface. Moreover, the etching rate depends on the origin of the c-Si wafer (Cz, Fz, etc.) [8], the wafer quality (defects, etc.) [8], the crystal orientation [18], and the doping concentration [20].

For laboratorial and industrial c-Si solar cells, a silicon base with a resistivity of ~1–3 Ω·cm is commonly used, which has been empirically found to provide a good balance between solar cell parameters. Therefore, a major number of literatures study the texturing process using Si wafers that have a resistivity of ~1 Ω·cm. However, decreasing base resistivity provides a way to increase *V*_oc_ and, accordingly, potentially the cell efficiency as well [21]. Brody et al. (2001) described the relation between the base resistivity of silicon solar cells and the cell efficiency, and they concluded that the optimal base resistivity should be lower or even much lower than the commonly used wafers.

In this paper, a texturing process on low-resistivity silicon wafers (~0.1 Ω·cm) in NaOH solution with the addition of IPA has been studied. The experiments were carried out with different IPA concentrations at 81.5°C (near the boiling point of IPA, 82°C) for different etching times, and the range of stirrer speeds (400 rpm) was involved. Detailed analyses of the surface phenomena, etching rates, surface morphology, and surface total reflectance have been carried out.

## 2. Experimental Materials and Methods

**Figure 3:**
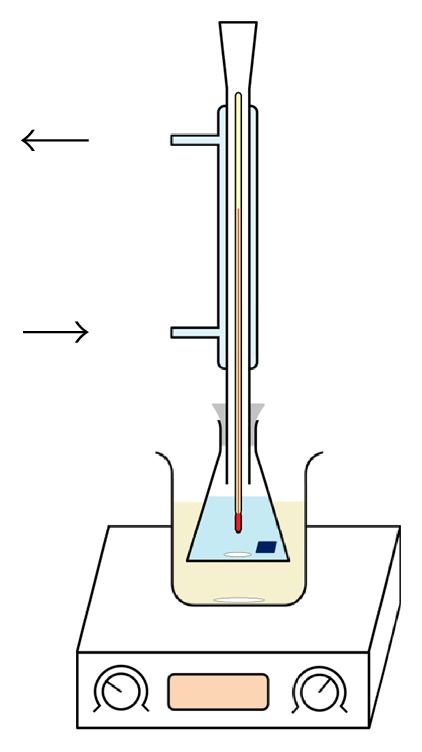
Schematic view of the texturing process setup used in this work.

The p-type monocrystalline Si 〈100〉 wafer with a thickness of 500 *µ*m and a resistivity of about 0.1 Ω·cm was used in this work. The silicon wafer was first cut to about 1 cm^2^ samples. The cleaning process was done in two steps: the first step was to remove contaminants from the silicon samples. In this step, the samples were cleaned in deionized water (DI-W) for 5 min followed by absolute ethanol for another 5 min under ultrasonic treatment at room temperature. The second step was to remove any native oxide. This step was carried out in ~10% HF for 1 min. After a thorough wash in flowing DI-W, the samples were etched in the solutions. The experiments were carried out in a glass flux dipped in an oil bath to achieve indirect heating of the solution (Figure 3).

**Figure 4:**
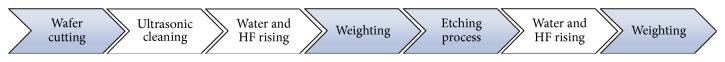
Schematic diagram of texturing wafer steps.

In addition, a water reflux condenser was put on the flux to recondense the vapor of chemicals (mainly that of the IPA) in order to keep the concentrations of the compounds constant. A thermometer with an accuracy of 0.1°C was inserted through the condenser to monitor the solution temperature. The alkaline compound used in this study was sodium hydroxide (NaOH) with concentration of 12% wt. dissolved in DI-W with a resistance of 18 MΩ. IPA was added to the solution with different volume concentrations (2, 4, 6, 8, and 10%). The samples were kept in the solution for different etching times ranging from 10 to 50 minutes and the temperature of the solution was controlled to be 81.5 ± 0.5°C. After the texturing process, the samples were washed by DI-W followed by 10% HCl (for 1 min) and 10% HF (for 30 sec) to remove any metallic impurities or silicon oxide. Finally, the wafers were washed again by DI-W (for 1 min) and were dried with an air jet. The etching rates were calculated from the weight difference of silicon samples after the texturing process using a microbalance. The steps of the texturing process are shown in Figure 4. IPA concentration (*C*_IPA_) and the time of etching (*t*_etch_) were varied in order to evaluate their effect on the pyramid construction and the total reflectance. The surface morphology of the silicon samples was analyzed firstly by an optical microscope, and then an atomic force microscope (AFM) was used to analyze the surface in detail.

To characterize the optical performance, a UV-Vis spectrophotometer (*CARY 5000*) with integrating sphere (*DRA-2500*) was used to measure the total surface reflectance in the wavelength range from 200 to 800 nm.

## 3. Results and Discussion

### 3.1. Etching Rate

**Figure 5:**
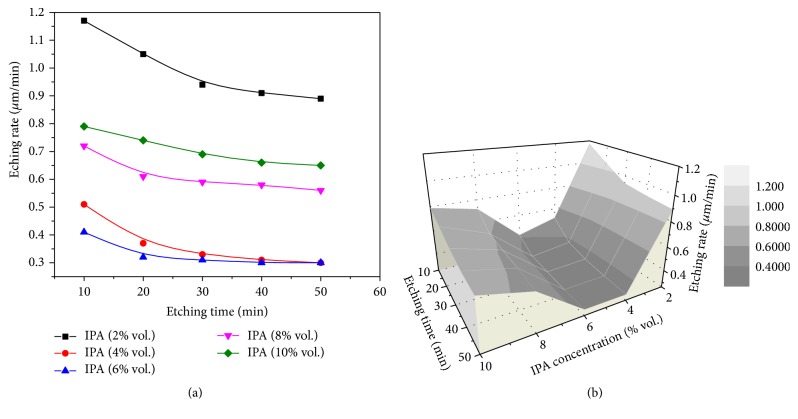
Etching rate as a function of etching time for different IPA concentrations: (a) 2D and (b) 3D.

The texturing process was carried out for the silicon samples in solutions of 12% wt. NaOH with various concentrations of IPA (2, 4, 6, 8, and 10% vol.) and for several etching times: 10, 20, 30, 40, and 50 min. In order to study the stability of the texturing process, the changes in the average etching rate with etching time and concentration of IPA were analyzed as shown in Figure 5.

**Figure 6:**
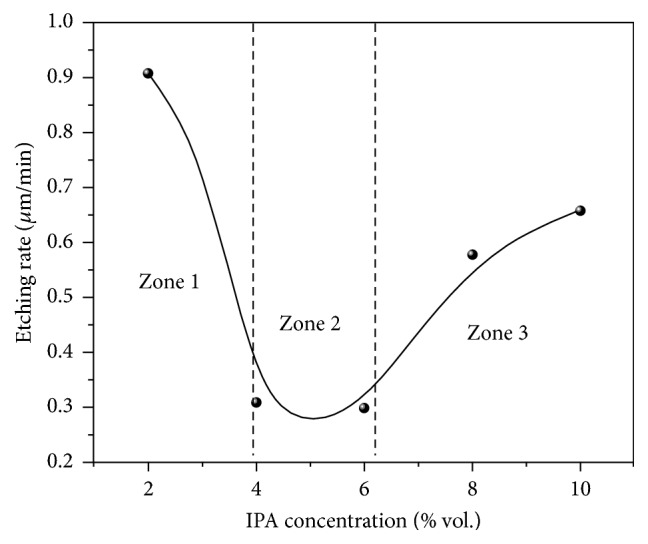
Etching rate as a function of IPA concentration at *t*_etch_ = 40 min.

It appears that *R*_etch_ decreases with increasing *t*_etch_. After 40 min, no further reduction of *R*_etch_ is observed in all solutions and this may be attributed to the strong dependence of the etching rate on the crystal orientation. At the beginning, solutions etch the Si wafer surface, that is, 〈100〉 orientation, with the highest etching rate. With time, 〈111〉 facets, which are etched with the lowest etching rate, are formed and all other orientations disappear.  Figure 6 shows *R*_etch_ as a function of *C*_IPA_ at *t*_etch_ = 40 min.

According to distinct features of *R*_etch_ versus *C*_IPA_, three different ranges can be defined. In the first range for *C*_IPA_ below 4%, *R*_etch_ decreases with increasing *C*_IPA_, and this behavior is due to the effect of IPA on the etching rate in alkaline solutions [5, 7].

In the second range for 4% < *C*_IPA_ < 6%, the average etching rate almost remains constant and goes through a minimum value, and this indicates that maximum anisotropic etching takes place in this range. In the third range for *C*_IPA_ higher than 6%, *R*_etch_ increases with increasing *C*_IPA_. This behavior is typical in etching Si wafer and can be attributed to the isotropic etching that starts to occur leading to a higher etching rate [7].

### 3.2. Optical Studies

**Figure 7:**
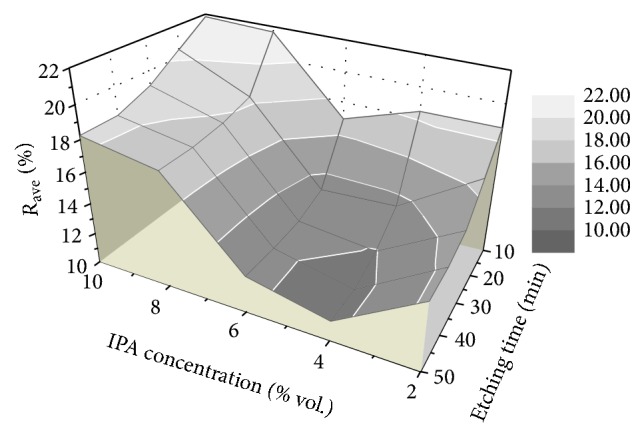
Average total reflectance as a function of etching time and IPA concentration.

**Figure 8:**
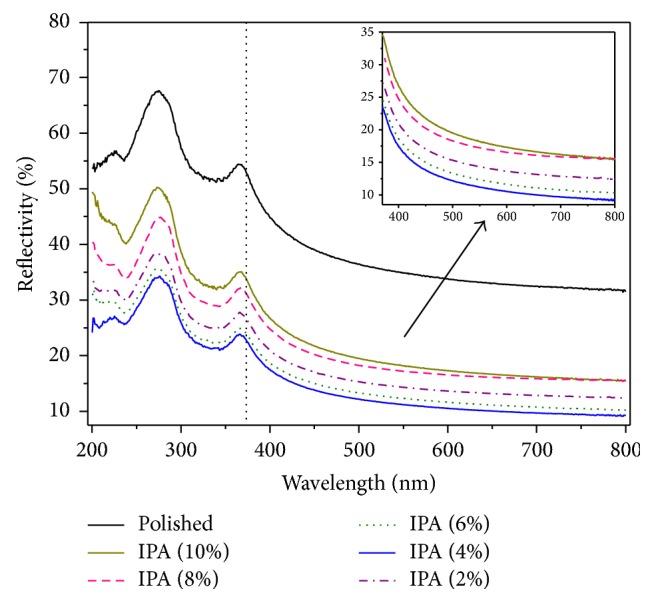
Total reflectance spectrum of textured samples for different IPA concentrations at *t*_etch_ = 40 min. The inset figure is a plot of *R*_ave_% versus *C*_IPA_ for the range 365 nm and above.

**Figure 9:**
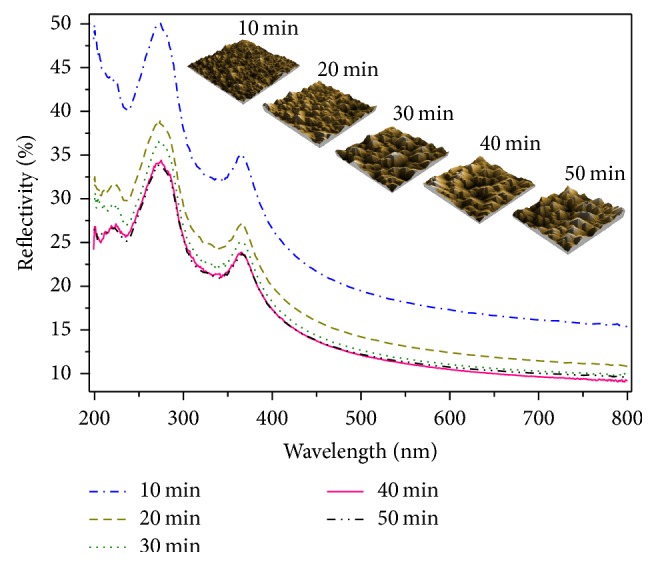
Total reflectance spectrum of textured samples for different etching times at *C*_IPA_ = 4% vol. The inset figure presents the AFM images for Si wafers' surface.

The total reflectance was used as a first check to identify the appropriate process parameters. It is worth noting that the specular reflectance is not a reliable parameter to check the effectiveness of the etching process. A low efficiency etching process can strongly decrease the specular reflection but increase the diffuse reflection, leaving the total reflectance almost unchanged. The total reflectance *R*% was recorded over the wavelength range 200–800 nm using integrating sphere. The changes in the average total reflectance *R*_ave_% (for the range 400 nm and above, see inset Figure 8) with *t*_etch_ and *C*_IPA_ were analyzed as shown in Figure 7. As we can see, *R*_ave_% decreases with increasing *t*_etch_. After 40 min, no further noticeable reduction of *R*_ave_% is observed in all samples. The total reflectance spectra (200–800 nm) of the samples for different *C*_IPA_ at *t*_etch_ = 40 min and for different *t*_etch_ at *C*_IPA_ = 4% were plotted in Figures 8 and 9, respectively. In general, we notice that the shoulder peak was obtained at 275 and 365 nm which are the peaks of silicon wafer. It was recorded in the wavelength range over 365 nm, the lowest average reflectance was 11.22%, and it was obtained for *C*_IPA_ = 4% vol. The reflectance values in the visible range were less than 17% and reached 9.1% at 800 nm. The average reflectance values are also summarized in Table 1.

### 3.3. Morphological Studies

**Figure 10:**
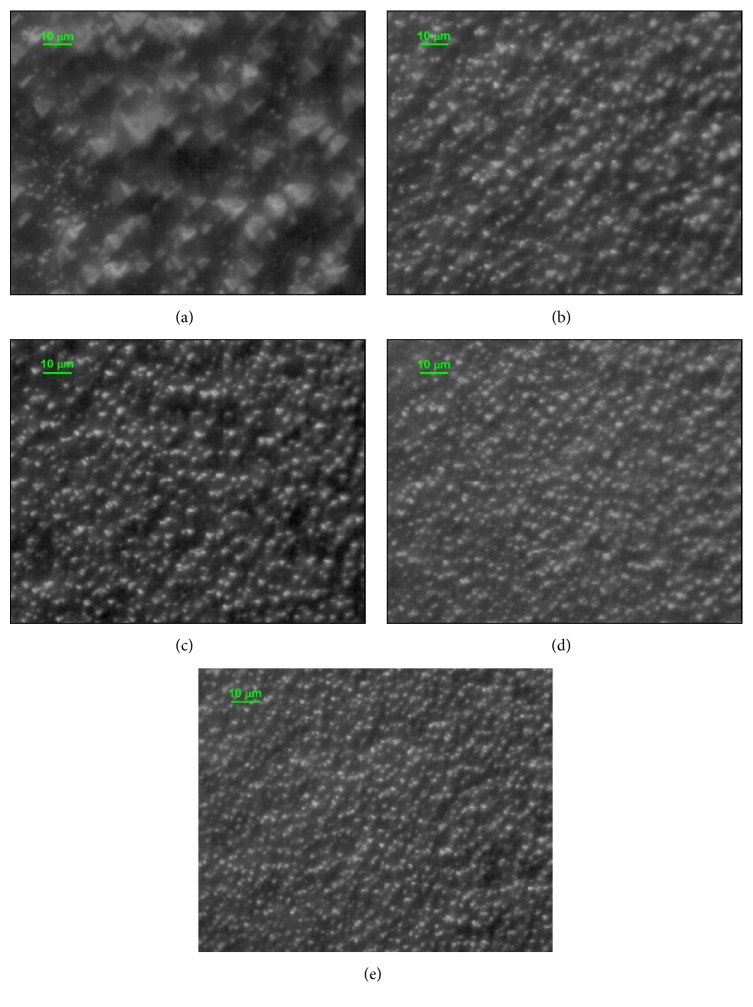
70 × 70 *μ*m^2^ optical microscope images for Si wafers' surface at different *C*_IPA_: (a) *C*_IPA_ = 2%, (b) *C*_IPA_ = 4%, (c) *C*_IPA_ = 6%, (d) *C*_IPA_ = 8%, and (e) *C*_IPA_ = 10%.

The density, the uniformity, and the size of the pyramids are important parameters in texturing silicon for solar cell production. The morphology of the wafer surface was analyzed using both an optical microscope and AFM.  Figure 10 shows the optical microscope images of the samples' surface under 600x magnification for different *C*_IPA_ at *t*_etch_ = 40 min.

For the sample etched with high IPA concentration (10%, Figure 10(e)), the surface is covered with small pyramids, the uniformity has been improved, and the specular reflectivity dominates. At low IPA concentrations (2%, Figure 10(a)), some pyramids with large size (~10 *µ*m) are formed, high size distribution is obtained (Figure 13(b)), the surface is not fully covered with pyramids, and the diffused reflectivity dominates.

**Figure 11:**
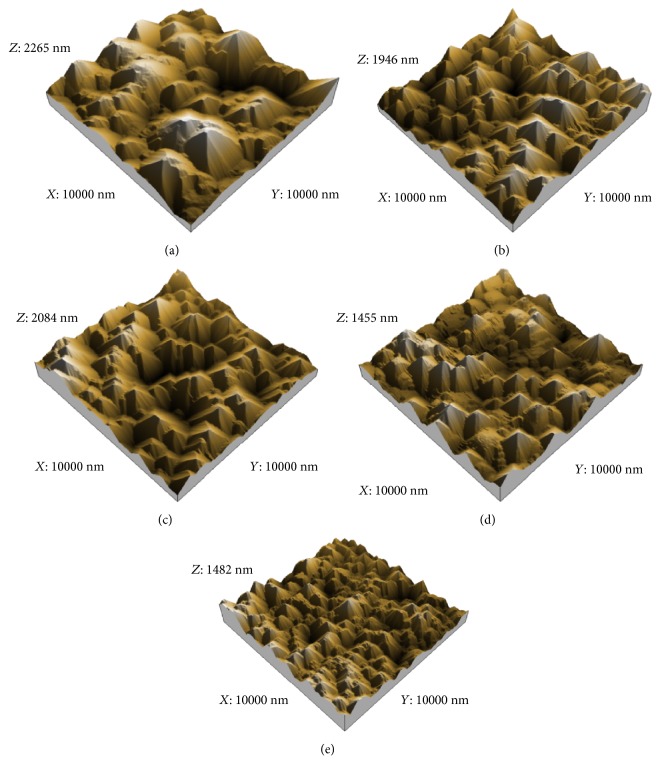
10 × 10 *μ*m^2^ AFM images for Si wafers' surface at different *C*_IPA_: (a) *C*_IPA_ = 2%, (b) *C*_IPA_ = 4%, (c) *C*_IPA_ = 6%, (d) *C*_IPA_ = 8%, and (e) *C*_IPA_ = 10%.

**Figure 12:**
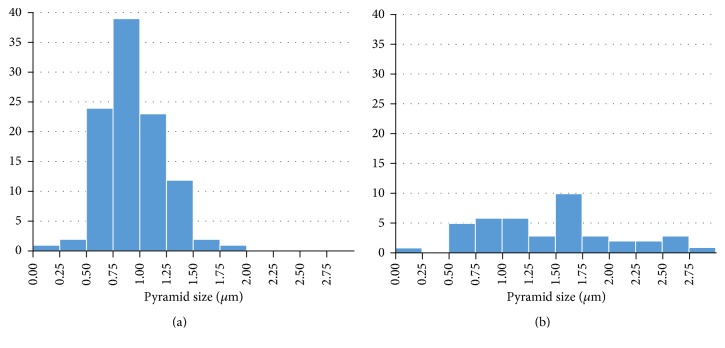
Pyramid size distribution for (a) *C*_IPA_ = 2% and (b) *C*_IPA_ = 10%.

**Figure 13:**
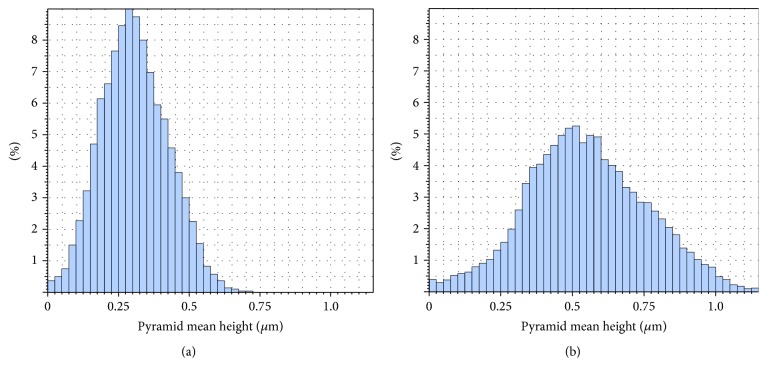
Pyramid height distribution for (a) *C*_IPA_ = 2% and (b) *C*_IPA_ = 10%.

The surface detailed topology of samples was examined using atomic force microscopy (AFM, Nanosurf easyScan2, Switzerland), tapping mode, and Tip Material-Si_3_N_4_ (silicon nitride). AFM images of the samples' surface for different *C*_IPA_ are shown in Figure 11. The impact of *C*_IPA_ on pyramid size, size, and height distribution (see Figures 12 and 13) is clearly highlighted in these images. The mean pyramid size (*S*_*m*_), root mean square (RMS) roughness, mean height (*h*_*m*_), and coverage ratio are summarized in Table 2.

As we show, for *C*_IPA_ = 2%, a low coverage ratio, high mean size, high mean pyramid height, and high surface roughness was obtained, whereas by increasing IPA concentration the coverage ratio increases and the mean pyramid size, mean height, and surface roughness decrease. According to Figures 12 and 13, the distributions of pyramid size and height were measured for low and high concentrations. A narrow distribution of pyramid size and height was observed at a lower concentration of 2%, while a broader distribution in the pyramid size and height was obtained at higher concentrations of 10%.

According to the morphological and reflectance results, we can safely conclude that the optimal pyramid coverage of the wafer surface, optimum pyramid size (~1.3 *µ*m), and total reflectivity (11.22%) are achieved around 4% IPA concentrations, and this result is in good agreement with the literatures [22].

## 4. Conclusion

The influence of IPA concentration and the etching time on the pyramidal surface structures was realized on etched mc-Si samples in alkaline solutions. Both *C*_IPA_ and *t*_etch_ were optimized based on the reflectance measurements. The optimization of the process variables yields the condition *C*_IPA_ = 4–6% vol. and *t*_etch_ = 40 min. The obtained surface was covered uniformly with ~1.3 *µ*m size pyramid structure and it has an average total reflectance of less than 11.22% on the visible range. These conditions have an optimal light trapping effect and are suitable for archiving the highest efficiency of solar cells compared to that with other etching conditions.

## Figures and Tables

**Table 1:** Average total reflectance for different IPA concentrations.

*C*_IPA_ (% vol.)	*R*_ave_% (400–800 nm)
2	14.40
4	11.22
6	12.34
8	17.45
10	18.25

**Table 2:** Texture parameters of Si wafers surface at different *C*_IPA_.

*C*_IPA_ (% vol.)	*S*_*m*_ (*μ*m)	RMS (*μ*m)	*h*_*m*_ (*μ*m)	Coverage ratio (%)
2	1.57	0.37	0.48	85.1
4	1.32	0.30	0.42	94.7
6	1.29	0.29	0.39	95.3
8	1.27	0.22	0.33	93.8
10	0.98	0.18	0.28	93.6
